# SAFE™ Initiative: A Step Closer to Positive Safety Culture and Improved Patient Experience

**DOI:** 10.7759/cureus.28554

**Published:** 2022-08-29

**Authors:** Cherie Plouff, Caitlin Byler, Melissa Hyatt, Claudine Jreissaty, Timisha Joe, April Thomas, Jennefer Mejicanos, Regina Smith, Braden R Hellstern, Victor J Hassid

**Affiliations:** 1 Oncology, The University of Texas MD Anderson Cancer Center, Houston, USA; 2 Plastic Surgery, The University of Texas MD Anderson Cancer Center, Houston, USA

**Keywords:** high reliability, quality improvement, patient reported experience, patient safety culture, oncology

## Abstract

Background

Positive safety culture is a key characteristic of a high-reliability organization; it is the leading service excellence standard and highest priority of The University of Texas MD Anderson Cancer Center. However, understanding the importance and impact of safety event reporting was limited at the MD Anderson campus in Sugar Land, Texas. Therefore, we implemented the Secure, Attentive, Focused, Engaged (SAFE™) initiative to create, foster, and continuously improve safety culture throughout the campus, with a secondary goal of impacting patient experience. Here, we review the SAFE™ initiative and its impact on our safety culture and patient experience.

Methods

The SAFE™ initiative was conceptualized and implemented in April 2017 by the leadership team at MD Anderson Cancer Center in Sugar Land. This initiative completely restructured our safety reporting and follow-up processes through leading by example, open safety meetings, transparent communication, emphasis on processes rather than people, and follow-up on any safety event entered or issue raised. We recorded quantitative measures and qualitative improvements, such as increased engagement and improved staff morale, using the results of institutional safety surveys in 2018 and 2020, which included a comparison to Agency for Healthcare Research and Quality's (AHRQ) national benchmarks and Press Ganey® patient experience scores. The AHRQ national benchmarks are based on the culture of safety surveys that measure staff views on safety, while the Press Ganey® patient experience scores measure patient perception. The SAFE™ initiative was then implemented at three additional MD Anderson Cancer Center campuses.

Results

During the data collection period of April 2017 to December 2021, we observed a sustained increase in safety event reporting at our campus, from 7.17 to 15.49 reports per month. We also observed a qualitative increase in safety meeting engagement and a higher participation rate in the institution-wide safety survey compared to MD Anderson Cancer Center overall. MD Anderson Cancer Center in Sugar Land scored above the national benchmarks in nine of the 13 domains in 2018 and all domains surveyed in 2020. Patient experience scores, measured by Press Ganey®, increased annually, with 2017, 2018, 2019, and 2020 fiscal year top box scores averaging 80.6%, 83.9%, 85.9%, and 86.4%, respectively.

Two of the additional locations showed improvement from 2018 to 2020 in the institution-wide Culture of Safety Employee Survey and scored above the AHRQ's national benchmarks in all the domains. The third location showed improvement from 2018 to 2020 on the institution-wide Culture of Safety Employee Survey in 11 of 15 domains and scored above the AHRQ's national benchmarks in all except one domain. The greatest improvements were error feedback, employee safety, and communication openness.

Conclusions

Positive safety culture is a requirement for a health care organization to be designated as a high-reliability organization. At MD Anderson Cancer Center in Sugar Land, we implemented an initiative that had a meaningful impact on the creation of a positive safety culture and was successfully scaled to additional locations.

## Introduction

The term "safety culture" and the phrase "high-reliability organization" can often be found within the same sentence or paragraph. This is no surprise, given the studied correlation between the two. The use of "safety culture" began outside of healthcare in organizations that are known for complex and hazardous work environments as part of their journey to becoming high-reliability organizations [1]. While there are several ways to define "safety culture," a general definition focuses on "an organization's shared perceptions, beliefs, values, and attitudes" that ultimately impact its commitment to safety and its effort to minimize harm [2]. As an organization striving for high reliability, The University of Texas MD Anderson Cancer Center has recognized a positive safety culture as a key characteristic of a high-reliability organization. Safety is also our leading service excellence standard and a core value; creating a positive safety culture is our top priority. Patient safety includes seven subcultures: leadership, teamwork, evidence-based, communication, learning, just culture, and patient-centered [3]. While safety culture is multi-faceted, there is growing evidence that a positive relationship exists between safety culture and patient experience [4,5].

MD Anderson Cancer Center is a multi-campus comprehensive cancer center with ambulatory clinics throughout the Houston area, Texas. Ambulatory care is delivered across five primary campuses. As recently as 2017, an understanding of the importance and impact of safety event reporting was limited at the MD Anderson Cancer Center campus in Sugar Land, Texas, a multidisciplinary ambulatory clinic offering medical, surgical, and radiation oncology, clinical services, such as infusion and radiation therapy, and laboratory, pharmacy, and rehabilitation services. Overall, the morale and culture around safety were negative: employees were hesitant to report safety events because of a lack of understanding of the reporting system, uncertainty about the next steps, fear of retaliation and punitive response to error, and a culture that made them feel as though they were "tattling" or being "tattled" on to management. Individual safe practices were evident, but we lacked a common thread that united us as a team on our journey toward zero patient harm.

A lack of understanding of the importance of a positive safety culture is an issue not isolated to our hospital - it is seen in hospitals worldwide. In a 2018 study, Okuyama, Galvao, and Silva completed a systematic review and meta-analysis that reviewed studies examining the hospital survey on patient safety culture. They found that the feeling of blame in hospital systems consistently had the least favorable responses, which negatively impacted staff reporting of safety issues and decreased the opportunity for correction. Leading factors for improving these areas across these studies included "effective communication, feedback following reporting, engaged leadership, and environments focused on learning from errors" [6].

To engage staff at all levels, create and maintain a positive safety culture, and improve the patient experience, MD Anderson Cancer Center in Sugar Land successfully developed the Secure, Attentive, Focused, Engaged (SAFE™) initiative to completely restructure our safety reporting and follow-up processes through leading by example, open safety meetings, transparent communication, emphasis on processes rather than people, and follow-up on any safety event entered or issue raised. We assessed the impact on our safety culture and patient experience by recording quantitative measures and qualitative improvements, such as increased engagement and improved staff morale, using the results of an institutional employee survey and Press Ganey® patient experience scores. These best practices have since been adopted throughout other locations across our institution. The results from this effort in 2017-2018 were presented at the American Society of Clinical Oncology Quality Care Symposium on September 28, 2018.

## Materials and methods

In early 2017, the leadership team at MD Anderson Cancer Center in Sugar Land conceptualized the SAFE™ initiative to improve our safety culture by improving engagement, eliminating the fear of retribution, and improving awareness and understanding of our reporting system and its purpose. This team included physician, advanced practice provider, nurse, business and administrative leaders. The components of the initiative included changes to our safety meeting structure, leadership members modeling positive safety practices, the development of a safety champion role, reviewing and integrating patient feedback related to safety, transparent reporting of and follow-up on safety events, increased and improved communication, and celebration of safety wins.

**Figure 1:**
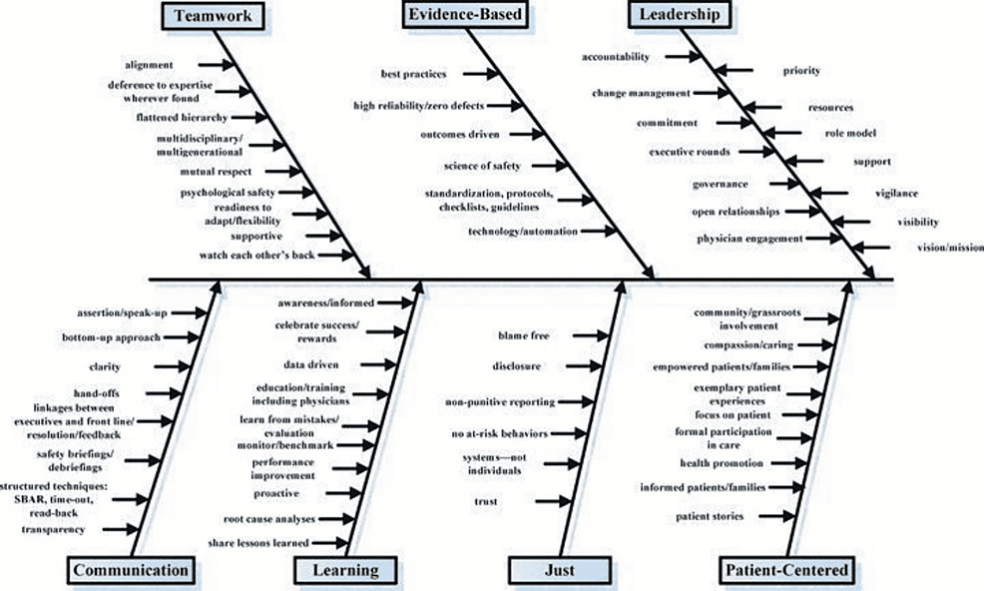
Hospital culture of patient safety Figure reproduced with permission from Sammer et al. [3].

As part of the initiative, over the course of several months, we identified best practices for each of the seven subcultures of patient safety (Figure 1). We also helped our team understand the positive outcomes of our safety culture, such as meaningful policy and practice changes that directly impact our patients, caregivers, and staff, and helped them understand that we were all working together to identify weak processes, not weak people.

We measured the results of our efforts using an institution-wide Culture of Safety Employee Survey in 2018, which was based on the Agency for Healthcare Research and Quality's (AHRQ) Hospital Patient Safety Culture Survey. We compared our results at MD Anderson Cancer Center in Sugar Land to those of the overall institution and AHRQ national benchmarks and correlated the results with Press Ganey patient® experience survey result trends. The AHRQ Hospital Patient Safety Culture Survey measures staff views on safety, while the Press Ganey® patient experience scores measure patient perception.

On the basis of these data, we shared our SAFE™ implementation, including best practices and lessons learned, with other MD Anderson Cancer Center regional campuses; we then compared our results with those of a two-year follow-up Culture of Safety Employee Survey in 2020 and Press Ganey® patient experience results in 2017, 2018, 2019, and 2020. Two new domains were included in the 2020 Culture of Safety Employee Survey (employee opinion survey pulse check and sexual misconduct) that were not accompanied by AHRQ national benchmarks.

## Results

MD Anderson Cancer Center in Sugar Land had 100% and 95% participation rates, respectively, in the 2018 and 2020 institution-wide Culture of Safety Employee Surveys, compared to the institution's 87% and 92% participation rates. In 2018, our campus scored higher than the AHRQ's national benchmarks on nine of the 13 domains surveyed (Table 1). When surveyed again in 2020, it scored above all of the national benchmarks on the domains surveyed. The greatest improvement from the 2018 Culture of Safety Employee Survey was in non-punitive response to error, and the highest score was in overall unit grade. After the SAFE™ initiative was shared across three other regional campuses, two locations showed improvement from 2018 to 2020 in the institution-wide Culture of Safety Employee Survey and scored above the AHRQ's national benchmarks in all of the domains. The third location showed improvement from 2018 to 2020 on the institution-wide Culture of Safety Employee Survey in 11 of 15 domains and scored above the AHRQ's national benchmarks in all except one domain. The greatest improvements were error feedback, employee safety, and communication openness.

**Table 1:** Culture of Safety Employee Survey results for MD Anderson Cancer Center in Sugar Land compared to the Agency for Healthcare Research and Quality's (AHRQ) national benchmarks (+) above benchmark; (-) below benchmark; YOY - year over year change; EOS - employee opinion survey

	Overall		Change (YoY)	National benchmark	
	2018	2020		2018	2020
Overall safety scores	76 (+)	74 (+)	-2	73	63
Management support for safety	76 (+)	80 (+)	4	70	70
Supervisor support for safety	76 (-)	83 (+)	7	77	78
Staffing	61 (+)	64 (+)	3	51	50
Learning from errors	82 (+)	85 (+)	3	71	71
Nonpunitive response to error	29 (-)	52 (+)	23	43	45
Communication openness	52 (-)	68 (+)	16	62	64
Feedback about error	67 (+)	85 (+)	18	66	68
Most employees report events	59	58 (+)	-1	-	46
Error reporting frequency	71 (+)	85 (+)	14	64	65
Teamwork within units	82 (+)	90 (+)	8	80	81
Teamwork across units	66 (+)	69 (+)	3	59	59
Handoffs	43 (-)	53 (+)	10	45	44
Unit grade	78	97 (+)	19	-	75
Perceptions of safety	65 (+)	76 (+)	11	63	64
Employee safety	75	87	12	-	-
EOS pulse check		74			
Sexual misconduct		87			

Since improving our safety culture at MD Anderson Cancer Center in Sugar Land, we have observed increasing patient experience scores, as measured by Press Ganey®. For the 2017, 2018, 2019, and 2020 fiscal years, our mean campus top box scores were 80.6%, 83.9%, 85.9%, and 86.4%, respectively.

**Figure 2:**
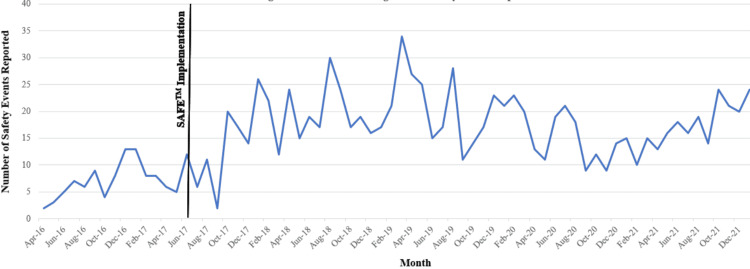
MD Anderson Cancer Center in Sugar Land reported safety events before and after SAFE™ implementation SAFE™ - Secure, Attentive, Focused, Engaged

**Figure 3:**
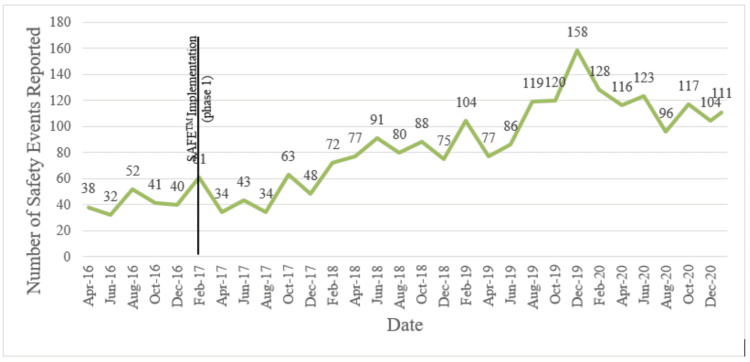
MD Anderson Cancer Center regional campus safety events before and after SAFE™ implementation SAFE™ - Secure, Attentive, Focused, Engaged

Prior to the implementation of SAFE™, the mean number of reported safety events on the Sugar Land campus was 7.17 per month. Since implementation, the results have demonstrated a mean of 15.49 reports per month through December 2021 (an increase of 110%; Figure 2). During the same time period, the number of overall reported events throughout MD Anderson's regional campuses increased by nearly 85% (Figure 3). We also observed a noticeable increase in attendance and participation in our biweekly safety meetings: most early meetings were attended by 10 or fewer individuals, while recent meetings have consistently included over 20 individuals. Further, a qualitative improvement in our safety culture was observed on our campus with the system-wide implementation of an anonymous reporting option in May 2019. Since that time, only four anonymous reports have been submitted, and only within the first two weeks of the option being available. Since June 2019, there has been no anonymous reporting. Staff willingness to submit safety events that are tied to their name indicates a positive safety culture in which individuals are not afraid to speak up and have a desire to be engaged in the process of making improvements.

## Discussion

The strengths of this study include the success of the scaled implementation at multiple locations and maintained employee engagement in safety practices. One limitation of this study is the focus on local initiatives, not accounting for concurrent institutional initiatives. While other institutional initiatives could account for some of the improvements seen in the employee survey and patient experience survey, the local data compared to institutional data support the significant impact of the SAFE™ initiative.

The identification of methods to achieve a positive safety culture can be challenging and time-consuming. As with most new projects and initiatives, the SAFE™ initiative has developed and improved over time, but it has resulted in a transformation from individual safe practices to a true safety culture at MD Anderson Cancer Center in Sugar Land, as evidenced by the comparison of our employee survey results compared to institutional results and national benchmarks, that will help us on our journey towards a high-reliability organization designation. The following sections represent areas that were studied and impacted during this initiative.

Leadership

Engaged leadership is a key factor in establishing an effective and lasting culture of safety within an organization. "Leaders play a critical role in improving the culture of safety by establishing the direction for change, aligning staff, and by motivating and inspiring" [7]. We identified early that morale surrounding safety culture starts within our leadership team. Initially, there was frequent frustration amongst the team because safety events were repeatedly discussed without a clear process or outcome. We improved leadership morale by separating our weekly safety event review from our weekly leadership meetings. It was important to have one meeting in which the sole focus was safety, as it not only provided dedicated time to review events and improvement strategies but also to discuss ideas and topics related to safety on our campus.

We also identified the need for commitment from all leadership team members to act as role models for the use of the safety reporting system. Clinical leaders began integrating safety reporting into their practices by sharing firsthand experiences with reporting safety events that caused any range of outcomes, from patient inconvenience to patient harm. Through leading by example, we were able to help identify issues that necessitated a safety report; we also walked staff through the process, if needed. We modeled and encouraged self-reporting; we also adjusted the way that we approached every safety event and the staff as we engaged in "studying" and "learning from," as opposed to "investigating" reported events. It gradually became a mutually accepted expectation that any time a system or process did not perform in an optimal way, a safety event would be entered.

Teamwork

Engagement is a critical component of developing a positive safety culture. Research shows that high levels of physician and employee engagement positively influence overall safety scores [8]. As a result of our initial leadership team safety meetings, we began engaging staff by identifying "Safety Champions" to serve on a Safety Committee. However, in early meetings, we identified a gap in expertise because we did not have representation from all areas during discussions of safety events. As a leadership team, we decided to convert our traditional Safety Committee to a committee that was open to all employees. This change helped create a culture of inclusivity and encouraged those with a vested interest to attend of their own volition and therefore be more engaged. It also helped encourage those who may not have the time to officially join a committee but would like to be involved as time allows, further broadening our reach and engagement.

In addition to the change in structure, we changed the meeting's frequency. Rather than holding a one-hour meeting once a month, we held 30-minute meetings once a week, which was later changed to bi-weekly. We observed a noticeable increase in meeting participation after making these changes; this allowed us to collaborate as a larger team and thus eliminate the separate weekly leadership event review. A document was created for use during this meeting that tracks the reported information and status of events.

During the initial phases of the initiative, some staff continued to show hesitation in openly sharing concerns or ideas. The leadership team continuously reinforced the idea that when it comes to safety, everyone has an equal voice and level of responsibility. While reporters were initially anonymous at our meetings, consistent support of all team members and an insistence on maintaining a respectful environment allowed us to change to open reporting with continued staff engagement.

Evidence-based

Including all team members in the discussion of safety events led to the increased identification of issues that required improvement and enriched discussion around evidence-based solutions. While working through and mapping processes that required standardization, we often needed to engage members of our business teams and ancillary services, further highlighting the importance of involving staff across all disciplines. This new model also supported the engagement of administrative and technology support staff by automating processes and improving electronic medical record challenges.

Communication

We obtained initial feedback on safety reporting and identified an opportunity to improve how follow-up was communicated to reporting staff members. On the basis of that feedback, we began directly communicating with employees every time they submitted an event so that we could recognize their commitment to safety, thank them for submitting the report, and encourage them to attend the safety meeting for further discussion. Having a mechanism in place for the reporter to share the event firsthand helped create a sense of ownership, involvement, and accountability, as well as an additional opportunity to recognize the reporter for their diligence. We then developed a monthly report that is emailed to all employees and includes the events from the previous month, along with the progress or resolution of each event.

We also improved communication by adding a safety topic to every team meeting as a reminder of the importance of safety to our journey toward becoming a high-reliability organization. To celebrate safety wins, we began hosting occasional "safety socials," providing sweets and discussion about recent improvements that were a direct result of safety events.

The early adoption of an open and positive safety culture was slow. We created a mechanism to communicate resolutions and successes so that all employees benefitted from the positive outcomes of a safety event review. We started keeping a list of positive changes and presenting them at each of our quarterly town halls. Finally, our monthly email newsletter began to include a "safety corner" in which we provided our event reporting metrics and a "great catch".

Learning

While the safety meetings and email summaries provide transparent communication, we wanted to take the learning opportunities a step further. When safety events are discussed that lead to the identification of a need for additional education or training or that should be shared as good reminders for colleagues, they are discussed in discipline-specific staff meetings, and new educational resources are created as needed.

Additional learning opportunities are created through annual safety fairs with participation from each discipline and department. Safety fair presentation topics have included radiation safety, security awareness, fall risks, and patient identification. Participation is encouraged by integrating presentations into annual goals and awarding the top posters on the basis of pre-determined criteria, as well as awarding a "People's Choice Award," voted on by peers.

Just culture

Organizations that promote a just culture empower employees to be proactive in monitoring workplace activities and become more involved in safety efforts [9]. Through improved leadership practices and morale surrounding safety culture, as well as celebrations of safety wins, employees began to trust that we were all working together to identify weak processes, not weak people. Over time, they were able to consistently observe that corrective actions were aimed at improving processes and that infrequent instances of disciplinary action were reserved for willful violations of policy, not human errors. These observations resulted in improved morale, which contributed to increased survey scores related to non-punitive response to error and employee willingness to openly report safety events. Further, we believe that the sustained increase in safety reporting was not a result of increased unsafe practices but rather an improvement in our safety culture and employee engagement in the process.

Patient-centered

Research by Press Ganey® shows that high performance on safety and quality measures can positively influence patient experience [8]. By having reporters and involved parties participate in the discussion of safety events, we are able to hear firsthand accounts of how these events directly affect patients. Team members are often able to convey a personal aspect during our meetings that is difficult to convey via a written report. We also implemented a process for obtaining feedback from patients through intermittent rounds. Comments and suggestions made by patients are discussed amongst the leadership team and at campus safety meetings.

## Conclusions

Safety is MD Anderson Cancer Center in Sugar Land’s leading service excellence standard and core value. It is also a key characteristic of a high-reliability organization, a designation that we strive to achieve as an organization. Further, an improved safety culture can positively influence the patient experience.

At the Sugar Land facility and, subsequently, at other MD Anderson Cancer Center campuses, we implemented an initiative that led to the creation of a positive safety culture and positively impacted patient experience metrics. We continue to assess and address limitations and challenges among our entire workforce with the ongoing goal of continuously improving our safety culture. The key components of this initiative include leading by example, open safety meetings, transparent communication, emphasis on processes rather than people, and follow-up on any safety event entered or issue raised. Through the SAFE™ initiative and its related practices, MD Anderson Cancer Center in Sugar Land has rapidly seen safety transform from individual safe practices to a true culture of safety in our journey toward high reliability. These transferrable ideas and best practices continue to be shared across our enterprise, as we recognize that maintaining a positive and just culture is a never-ending journey that requires consistent attention to detail and engagement by all team members. We continue to optimize our safety practices and use patient safety and patient experience surveys to monitor performance.
